# The impact of teeth and dental restorations on gray value distribution in cone-beam computer tomography: a pilot study

**DOI:** 10.1186/s40729-023-00493-z

**Published:** 2023-09-07

**Authors:** Oliver Wagendorf, Susanne Nahles, Kirstin Vach, Florian Kernen, Stefan Zachow, Max Heiland, Tabea Flügge

**Affiliations:** 1 Department of Oral and Maxillofacial Surgery, Charité, Universitätsmedizin Berlin, Corporate Member of Freie Universität Berlin, Humboldt-Universität zu Berlin and Berlin Institute of Health, Augustenburger Platz 1, 13353 Berlin, Germany; 2 Faculty of Medicine and Medical Center, Institute of Medical Biometry and Statistics, University of Freiburg, Stefan-Meier-Straße 26, 79104 Freiburg im Breisgau, Germany; 3 Department of Oral and Maxillofacial Surgery and Translational Implantology, Faculty of Medicine and Medical Center, University of Freiburg, Stefan-Meier-Straße 26, 79104 Freiburg im Breisgau, Germany; 4 Zuse Institute Berlin (ZIB), Takustraße 7, 14195 Berlin, Germany

**Keywords:** CBCT, Gray values, Bone density, Oral surgery

## Abstract

**Purpose:**

To investigate the influence of teeth and dental restorations on the facial skeleton's gray value distributions in cone-beam computed tomography (CBCT).

**Methods:**

Gray value selection for the upper and lower jaw segmentation was performed in 40 patients. In total, CBCT data of 20 maxillae and 20 mandibles, ten partial edentulous and ten fully edentulous in each jaw, respectively, were evaluated using two different gray value selection procedures: manual lower threshold selection and automated lower threshold selection. Two sample *t* tests, linear regression models, linear mixed models, and Pearson's correlation coefficients were computed to evaluate the influence of teeth, dental restorations, and threshold selection procedures on gray value distributions.

**Results:**

Manual threshold selection resulted in significantly different gray values in the fully and partially edentulous mandible. (*p* = 0.015, difference 123). In automated threshold selection, only tendencies to different gray values in fully edentulous compared to partially edentulous jaws were observed (difference: 58–75). Significantly different gray values were evaluated for threshold selection approaches, independent of the dental situation of the analyzed jaw. No significant correlation between the number of teeth and gray values was assessed, but a trend towards higher gray values in patients with more teeth was noted.

**Conclusions:**

Standard gray values derived from CT imaging do not apply for threshold-based bone segmentation in CBCT. Teeth influence gray values and segmentation results. Inaccurate bone segmentation may result in ill-fitting surgical guides produced on CBCT data and misinterpreting bone density, which is crucial for selecting surgical protocols.

**Graphical Abstract:**

**Figure:**
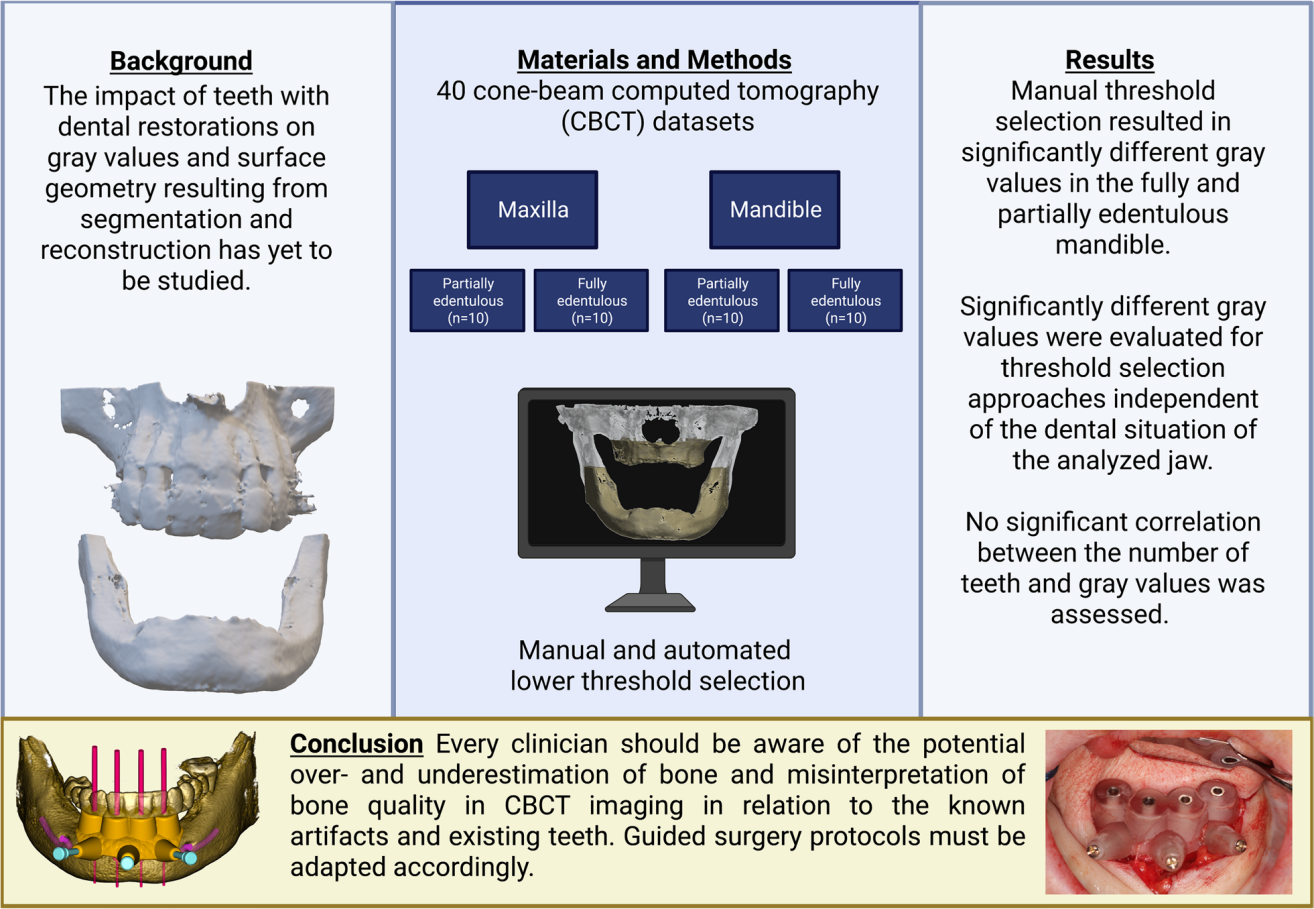
Created with BioRender.com

**Supplementary Information:**

The online version contains supplementary material available at 10.1186/s40729-023-00493-z.

## Background

Cone-beam computed tomography (CBCT) is dentistry’s most commonly used tool for three-dimensional display of the facial skeleton [1] In implant dentistry and craniomaxillofacial surgery, CBCT plays a significant role in preoperative planning, visualization, assessment of bony structures, and surgical guide generation [2, 3].

For the computer-assisted visualization of anatomy and the design and preoperative production of surgical tools (e.g., drill guides), CBCT data are used in conjunction with specific software. It is used for preoperative visualization and treatment planning, virtual surgery, and CAD/CAM (computer-aided design/computer-aided manufacturing) guides for resection and reconstruction [4].

Medical image data typically are two- or three-dimensional regular scalar fields, where each scalar value is represented by a picture/volume element (pixel/voxel). Each volume element (voxel) has a specific gray value, representing the tissue density it captures. In CT imaging, such scalar values represent radiodensity measured in Hounsfield Units (HU). Based on these values, tissues of different radiopacity can be discriminated. A three-dimensional representation of the imaged volume is visualized by selecting a range of gray values/HU. Only voxels in that particular range and specific anatomical structures (e.g., bone) are displayed by choosing an upper and a lower threshold for gray values [5]. The procedure of assigning a label to particular structures is defined as binary segmentation. The respective segment of the data volume can be converted into a surface representation by extracting the boundaries of the segment via triangulation [6]. Such a surface model is often used for treatment planning [7].

The more contrast an image displays, the easier is the segmentation of anatomical structures, whereas minor differences in density among adjacent structures complicate segmentation. A large variety of factors such as tube voltage, tube current, detector type, voxel size, the field of view (FOV), and reconstruction algorithm, respectively, may cause a shift of density values of the complete volume in either direction [8]. Furthermore, image artifacts in the form of noise, scatter, extinction, beam hardening, exponential edge gradient effect, aliasing, ring artifacts, and moving artifacts result in density values not correlating with anatomical structures. [9] Default values transferred from CT imaging may not apply to CBCT [10–12]. A transfer of gray values into pseudo-Hounsfield units was proposed. However, the diagnostic capacity of this procedure for clinical image data is missing [12]. The most commonly used segmentation algorithms in clinical routine are semi-automatic, with a standard upper and lower threshold for gray values (global thresholding) and a user's input for surface model generation [13, 14].

In a literature review by van Eijnatten et al., a trained clinician's global thresholding with laborious post-processing showed the highest accuracy [13]. Due to the remaining inaccuracies, more advanced techniques have been developed to gain accurate segmentations without manual post-processing. Fully automated segmentation algorithms provide comparable results to trained clinicians using a defined gray value range and automated threshold selection [15, 16]. These techniques, however, have not been implemented in commercially available clinical treatment planning software [13].

The accuracy of the segmented bone surface is crucial for the fit of a drill guide and the transfer of a virtually planned surgery to the intraoperative site [17–20]. The potential error introduced by bone segmentation and surface reconstruction could lead to a poor fit of the drill guide and consecutive harm to vital anatomical structures or a misinterpretation of the bone density, resulting in an inappropriate drilling protocol and consecutive missing primary stability of dental implants. Whereby the influence of dental implants on gray values in CBCT is widely known and studied, to the best of our knowledge, there is no study evaluating the impact of teeth with dental restorations on the resulting distribution of gray values in CBCT images and thereby, surface geometry resulting from segmentation and surface reconstruction [21–23]. Existing teeth may influence the overall gray value distribution in the data set by adding a diverse number of gray values, representing enamel, dentin, and dental restorations, which may lead to a shift in the constitution of the data set.

This study aimed to compare different bone segmentation thresholds from different methods in fully and partially edentulous patients to investigate if present teeth with dental restorations influence gray value distribution and, therefore, the geometry of surface models resulting from segmentation.

The study's primary outcome is to determine whether the grey value distribution in CBCT is influenced by the presence of teeth with dental restorations or other foreign bodies (e.g., radiographic stents). The secondary outcome is the impact of manual and automated threshold selection on segmentation. The study aims to evaluate whether there is a statistically significant difference in grey values between CBCT scans with and without teeth or foreign bodies and assess these structures' impact on bone segmentation.

The study aims to test the null hypothesis that teeth or other foreign bodies do not influence the grey value distribution in CBCT.

## Materials and methods

### Data set selection

**Fig. 1:**
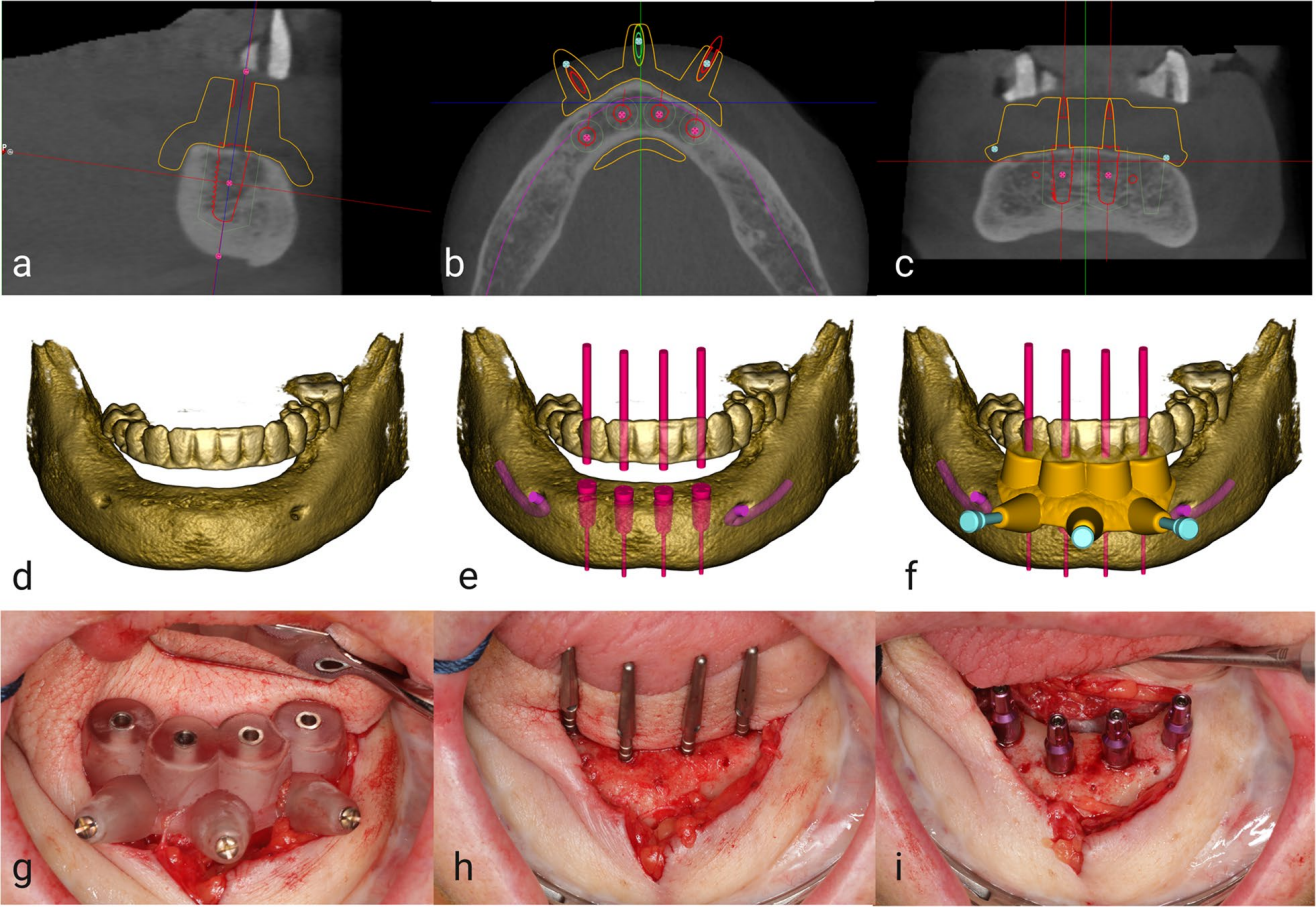
Case series of a bone-supported surgical guide, designed and fabricated using CBCT. Outline of the surgical guide and CBCT in sagittal (**a**), axial (**b**), and coronal (**c**) plane. Mandibular segmentation with prosthetic setup (**d**), implant position (**e**), and 3D surgical guide (**f**). Clinical images of implant placement with the surgical guide in situ (**g**), drilled implant positions with direction markers (**h**), and implants in situ (**i**)

The study protocol complied with the STROBE guidelines and was approved by the Ethics Committee of the Charité—Universitätsmedizin Berlin, Germany (EA4/111/21). It conforms to the Declaration of Helsinki and the European Medicines Agency Guidelines for Good Clinical Practice. Forty CBCT data sets of partially and fully edentulous jaws acquired between January 2018 and February 2021 for dental implant planning were selected. (Fig. 1) Thereof, twenty upper and twenty lower jaws, ten edentulous and ten partially edentulous jaws, respectively. Partially edentulous jaws were assigned to Kennedy classes 1 and 2, and the number of remaining teeth and dental restorations were recorded, regardless of their size. All CBCT scans were standardized using ProMax 3D Max, Pro Face Med Series H23 120 kV (SCS Sophisticated Computer tomographic Solutions GmbH, Aschaffenburg, Germany) with 120 kV tube voltage and 5 mA tube current, isotropic voxel size of 0.2 mm, bit depth 16 using a Field of View of 20 × 10 cm and exported in DICOM format.

### Segmentation procedure

Bone segmentation was performed with manual threshold selection, automated threshold selection of the complete data set, and automated threshold selection of the region of interest, respectively, using Amira ZIBEdition (Version 2021.27, https://amira.zib.de/). All segmentations were performed on a reporting monitor (MX217-HB, EIZO Europe GmbH, Germany) under the same lighting conditions (RK 5 due to DIN 6868-157) by the same investigator (OW) after calibration for adequate segmentation results. All manual segmentations were performed twice witch in a distance of 3 months. The data sets were navigated in corono-axial direction. The mean of both selected manual selected thresholds was used for further analysis. Intra-examiner reproducibility was 99,4%.

**Fig. 2:**
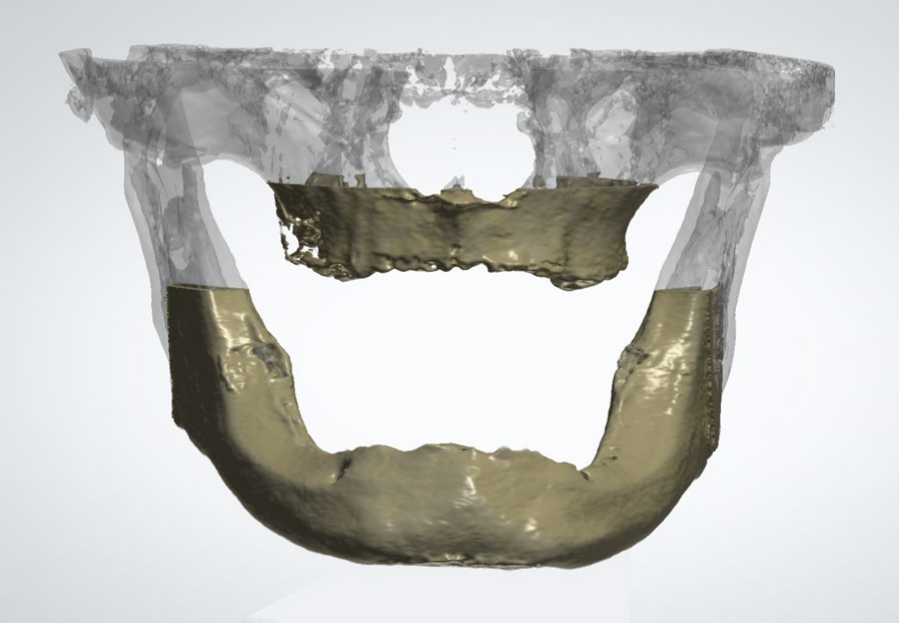
Visualization of the chosen region of interest (ROI) in the maxilla and mandible within the complete data set as an example in one data set

For manual threshold selection, the CBCT data set was cropped to the region of interest (ROI), the tooth-bearing maxilla or mandible, by visual inspection (Fig. 2). A global threshold was manually selected by visual inspection of the resulting iso-surface and marked areas in coronal and axial slices. The chosen lower threshold value was used for further analysis.

For automated threshold selection using the complete CBCT, the gray value range was limited from -500 to 1250 to generate a nearly bimodal gray value histogram and calculate a lower threshold value [16]. The resulting segmentation was inspected visually, and the selected lower threshold value was used for further analysis.

**Fig. 3:**
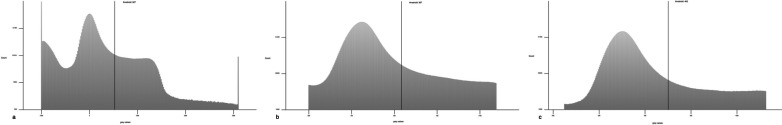
Example histograms for manual and automated lower threshold selection in the complete CBCT and ROI, edentulous mandible. Showing the total spectrum of the gray values in CBCT in manual lower threshold selection (**a**) and the limited spectrum of gray values from -500 to 1250 in automated threshold selection in complete CBCT (**b**) and ROI (**c**). Selected threshold for each procedure is marked

For automated threshold selection of the region of interest, the image was cropped to the ROI, the maxilla or the mandible, respectively. The threshold selection process was similar to the previously described in the complete CBCT volume. The resulting 120 different lower threshold values were used for further statistical analysis (Fig. 3).

### Statistical analysis

Two sample *T* tests were used to compare threshold values of partially and total edentulous jaws in both maxilla and mandible. Linear regression models were applied to evaluate the influence of the opposite jaw on the threshold results of each segmentation procedure within each jaw type (maxilla, mandible). Linear mixed models were applied to compare the results of the different segmentation procedures in each jaw. For paired subsequent tests, the method of Scheffe was used to correct for multiple testing. Pearson's correlation coefficients were computed to evaluate an association of the number of teeth and dental restorations and gray values for each segmentation procedure. The level of significance was set at *α* = 0.05. STATA software (Release 17, StataCorp LLC, College Station, TX, USA) was used for statistical analysis.

## Results

The remaining number of teeth in the partially edentulous patients and the teeth in the opposing for each patient are shown in Table 1 for the ROI maxilla and Table 2 for the ROI mandible.

**Table 1:** Fully and partially edentulous patients in the maxilla with remaining teeth, restorations, restorations material and opposing jaw remaining teeth, restorations and restorations material

Patient	Edentoulus	Opposite jaw	Teeth analysed jaw	Restaurations analysed jaw fillings	Analysed jaw fillings composite	Analysed jaw fillings metal	Restaurations analysed jaw fixed restorations	Analysed jaw fixed restorations ceramics	Analysed jaw fixed restorations metal	Teeth opposing jaw	Restaurations opposing jaw fillings	Opposing jaw fillings composite	Opposing jaw fillings metal	Restaurations opposing jaw fixed restorations	Opposing jaw fixed restorations ceramics	Opposing jaw fixed restorations metal	
1ed	Total	Partial	0	0	0	0	0	0	0	10	0	0	0	14	0	14	
2ed	Total	Edentulous	0	0	0	0	0	0	0	0	0	0	0	0	0	0	
3ed	Total	Partial	0	0	0	0	0	0	0	10	0	0	0	1	1	0	
4ed	Total	Edentulous	0	0	0	0	0	0	0	0	0	0	0	0	0	0	
5ed	Total	Partial	0	0	0	0	0	0	0	9	0	0	0	0	0	0	
6ed	Total	Partial	0	0	0	0	0	0	0	4	0	0	0	4	0	4	
7ed	Total	Edentulous	0	0	0	0	0	0	0	0	0	0	0	0	0	0	
8ed	Total	Edentulous	0	0	0	0	0	0	0	0	0	0	0	0	0	0	
9ed	Total	Edentulous	0	0	0	0	0	0	0	0	0	0	0	0	0	0	
10ed	Total	Partial	0	0	0	0	0	0	0	6	0	0	0	0	0	0	
1pa	Partial	Partial	10	0	0	0	0	0	0	6	1	1	0	0	0	0	
2pa	Partial	Partial	7	2	2	0	5	2	3	4	1	1	0	3	0	3	
3pa	Partial	Full	11	1	1	0	4	2	2	14	1	1	0	5	0	5	
4pa	Partial	Partial	2	0	0	0	2	0	2	8	0	0	0	7	0	7	
5pa	Partial	Full	7	2	2	0	0	0	0	15	9	6	3	1	0	1	
6pa	Partial	Partial	7	6	2	4	1	1	0	10	2	0	2	3	0	3	
7pa	Partial	Edentoulus	9	0	0	0	9	0	9	0	0	0	0	0	0	0	
8pa	Partial	Partial	5	0	0	0	3	0	3	3	0	0	0	3	0	3	
9pa	Partial	Full	10	2	2	0	4	0	4	14	3	3	0	4	0	4	
10pa	Partial	Partial	7	1	0	1	3	3	0	7	1	1	0	1	1	0

**Table 2:** Fully and partially edentulous patients in the mandible with remaining teeth, restorations, restorations material and opposing jaw remaining teeth, restorations and restorations material

Patient	Edentoulus	Opposite jaw	Teeth analysed jaw	Restaurations analysed jaw fillings	Analysed jaw fillings composite	Analysed jaw fillings metal	Restaurations analysed jaw fixed restorations	Analysed jaw fixed restorations ceramics	Analysed jaw fixed restorations metal	Teeth opposing jaw	Restaurations opposing jaw fillings	Opposing jaw fillings composite	Opposing jaw fillings metal	Restaurations opposing jaw fixed restorations	Opposing jaw fixed restorations ceramics	Opposing jaw fixed restorations metal	
1ed	Total	Edentulous	0	0	0	0	0	0	0	0	0	0	0	0	0	0	
2ed	Total	Edentulous	0	0	0	0	0	0	0	0	0	0	0	0	0	0	
3ed	Total	Edentulous	0	0	0	0	0	0	0	0	0	0	0	0	0	0	
4ed	Total	Edentulous	0	0	0	0	0	0	0	0	0	0	0	0	0	0	
5ed	Total	Edentulous	0	0	0	0	0	0	0	0	0	0	0	0	0	0	
6ed	Total	Edentulous	0	0	0	0	0	0	0	0	0	0	0	0	0	0	
7ed	Total	Partial	0	0	0	0	0	0	0	6	0	0	0	11	0	11	
8ed	Total	Partial	0	0	0	0	0	0	0	9	0	0	0	9	0	9	
9ed	Total	Partial	0	0	0	0	0	0	0	3	0	0	0	3	0	3	
10ed	Total	Edentoulus	0	0	0	0	0	0	0	0	0	0	0	0	0	0	
1pa	Partial	Edentulous	6	0	0	0	2	0	2	0	0	0	0	0	0	0	
2pa	Partial	Partial	8	0	0	0	7	0	7	2	0	0	0	2	0	2	
3pa	Partial	Partial	3	0	0	0	3	0	3	5	0	0	0	3	0	3	
4pa	Partial	Edentulous	10	0	0	0	1	1	0	0	0	0	0	0	0	0	
5pa	Partial	Edentulous	9	0	0	0	0	0	0	0	0	0	0	0	0	0	
6pa	Partial	Partial	4	1	1	0	3	0	3	7	2	2	0	5	2	3	
7pa	Partial	Partial	6	1	1	0	0	0	0	10	0	0	0	0	0	0	
8pa	Partial	Edentulous	6	0	0	0	0	0	0	0	0	0	0	0	0	0	
9pa	Partial	Partial	10	2	0	2	3	0	3	7	6	2	4	1	1	0	
10pa	Partial	Partial	7	1	1	0	1	1	0	7	1	0	1	3	3	0	

The gray values for each manual and automated selection for the maxilla and mandible are displayed in the Additional file 1: Tables S1 and S2.

**Fig. 4:**
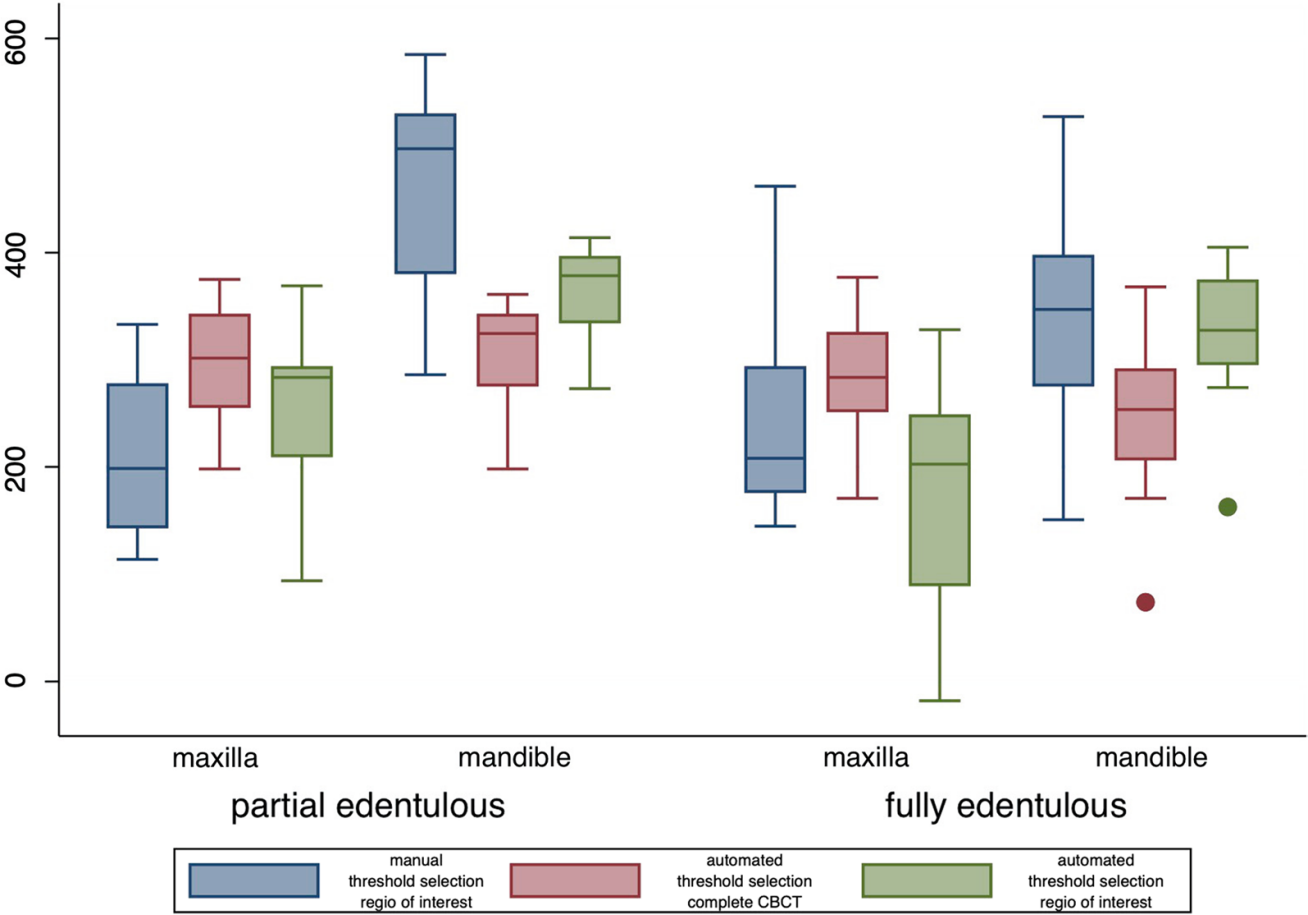
Distribution of gray values in each jaw and different segmentation procedures

The distribution of lower threshold values for each jaw and all segmentation procedures are shown in Fig. 4. Mean values and standard deviations for each jaw and all segmentations procedures are shown in Table 3.

**Table 3:** Mean gray values and standard deviation (SD) in each jaw and different segmentation procedures

Location	Edentulous maxilla	Partial edentulous maxilla	Edentulous mandible	Partial edentulous mandible
Manual threshold selection	242 (31)	211 (24)	345 (33)	476 (30)
Automated threshold selection complete data set	286 (20)	297 (17)	248 (28)	306 (16)
Automated threshold selection cropped data set	181 (34)	256 (25)	322 (22)	359 (16)

Two-sample *t* tests showed no significantly lower threshold values for manual threshold selection in partially compared to fully edentulous maxillae. Significantly different gray values were found for partially and fully edentulous mandibles (*p* = 0.0088), with lower gray values in fully edentulous mandibles (difference 130). Automated threshold selection tends to lower gray values in fully compared to partially edentulous mandibles, in the complete CBCT (*p* = 0.087, difference 58) and maxilla, in the cropped CBCT data set (*p* = 0.090, difference 75) could be found. The opposing jaw did not have a significant influence on gray values.

The comparison of segmentation procedures in the maxilla showed a significant difference in automated threshold selection of the complete CBCT vs. manual threshold selection (*p* < 0.001) with higher values in automated selection (difference: 65). Automated threshold selection of the complete CBCT resulted in significantly higher threshold values than the cropped CBCT (*p* < 0.001, difference 73). No significant difference was shown between manual and automated threshold selection in the cropped data set. In the mandible significant differences could be shown in all comparisons: automated complete CBCT vs. manual (*p* < 0.001, difference: 133), automated cropped CBCT vs. manual (*p* = 0.004, difference: 67) and automated complete CBCT data set vs. cropped data set (*p* = 0.007, difference: 64).

The comparison of automated threshold selection in the cropped data set and manual threshold selection resulted in significantly lower gray values in fully edentulous maxillae (*p* = 0.001 difference: 62) and partially edentulous mandibles (*p* < 0.001, difference: 110). Conversely, in partially edentulous maxillae, significantly higher gray values were observed (*p* = 0.009, difference: 49). Both threshold selection methods resulted in no significant differences in edentulous mandibles.

**Fig. 5:**
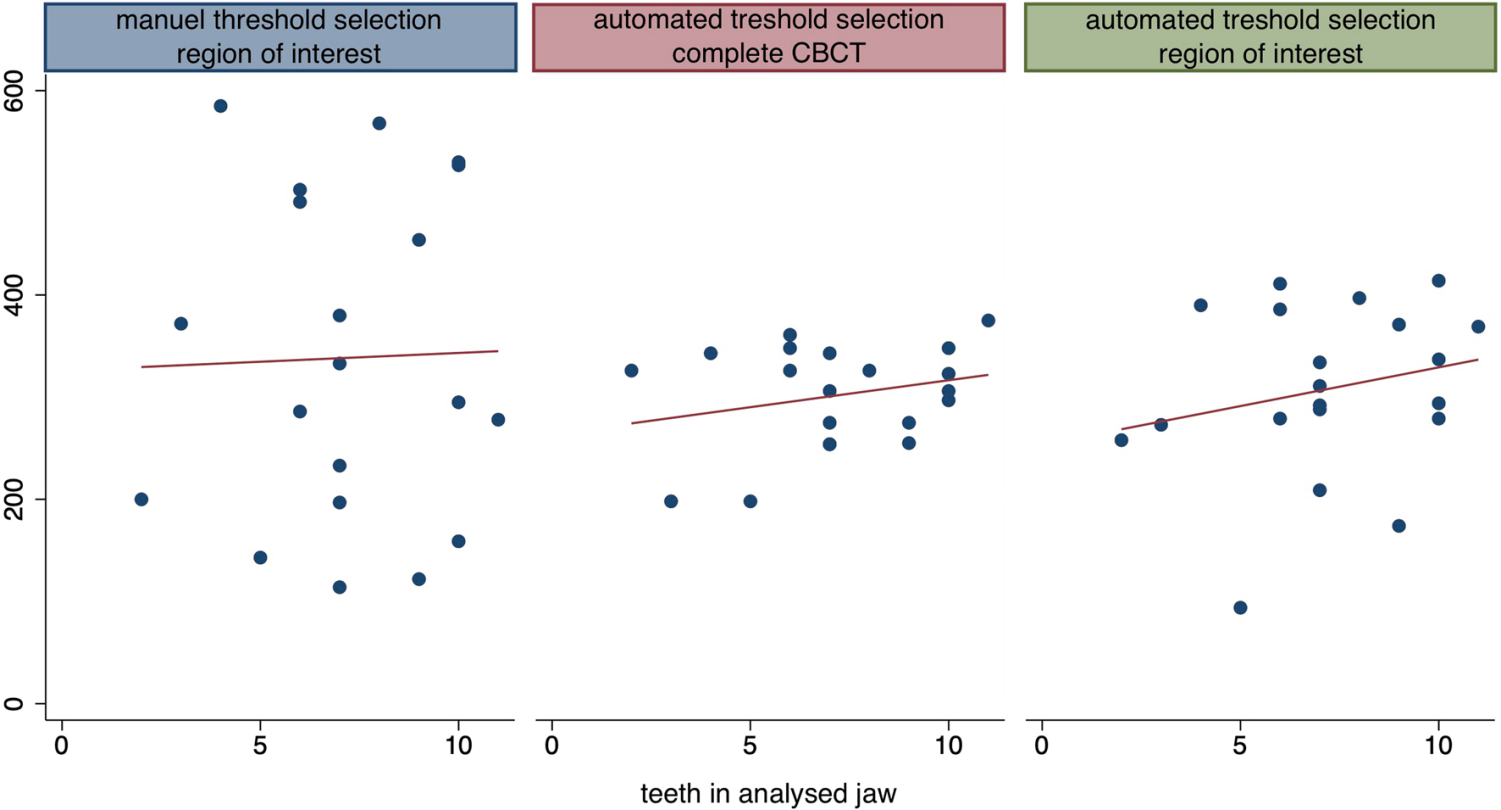
Association between gray values and number of teeth in partially edentulous jaws. The red line shows the result of the linear regression analysis

Correlation for evaluating the number of teeth and gray values selected by the different procedures showed no significant correlations but slight tendencies to a higher gray value in patients with more teeth for automated segmentation (Fig. 5). The restorations material showed no significant correlation to higher gray values.

## Discussion

The selected threshold levels were significantly different for the maxilla and mandible and the three threshold selection procedures. Teeth influenced the selected gray values for the segmentation of bone. These effects were not only present around the teeth but in the whole data set, reflected in threshold values for segmentation. The influence of teeth on gray value distribution was more substantial for automated threshold selection and lower for manual threshold selection.

Gray values represent the tissue in each voxel and are used for density measurements of bone and surface model creation and display. An accurate surface model of craniofacial bone is crucial for digital surgery planning and guide production [24]. Manual threshold selection and a modified way for objective threshold selection are the most widely used techniques for surface model creation and exhibit good clinical results. However, they require individual input [13, 15].

To evaluate the influence of teeth on grey values, partially edentulous patients were selected according to the average number of remaining teeth. An average of eight teeth in the maxilla and nine in the mandible for patients between 65 and 74 years in Germany was presumed [25]. The included patients had an average of 7.5 teeth in the maxilla and 6.9 teeth in the mandible, representing the demographic distribution. To the author's knowledge, the number and distribution of teeth were previously not regarded to analyze gray values in CBCT. The former studies focused on the influence of metal restorations and dental implants on the surrounding gray values in vitro and in vivo; however, the impact of teeth on the complete data set has not been investigated [21–23].

Different segmentation methods, threshold levels, and acquisition specifications on (CB)CT-derived surface models were previously evaluated, and optical scans were used as a reference to assess accuracy in vitro and in vivo [5, 13–15, 26–34]. A literature review of different image segmentation methods used for medical imaging data reported deviations of 0.04 mm to 1.9 mm between surface models. The most used method, global threshold selection, resulted in deviations below 0.6 mm and implied extensive manual post-processing [13]. Standard values for bone segmentation of the whole skull and manual global threshold selection deviated − 2.3 mm to 4.8 mm from optical scans of cadavers [5]. Deviations between models created with manual global threshold selection of the mandible by an experienced clinician and laser surface scans of the cadaver mandible were 0.76 mm ± 0.39 mm [14]. Intra- and inter-examiner deviations of surfaces derived from manual threshold selection were reported with 0.18 mm and 0.15–0.26 mm, respectively [33]. Surface deviations of 0.6 mm were reported for models created with manual and automated threshold selection [15].

Due to the variances between the different image segmentation methods, manual global threshold selection, the most used method in medical image segmentation, and automated threshold selection were used in this study. All manual threshold selections were performed once, by one experienced clinician, under the same circumstances, without blinding. Automated threshold selection did not require individual input from the examiner and was adopted in the presented study as previously described [15]. The threshold range from -500 to 1250 was set based on Misch et al. and Norton et al. to transform the original histogram into a bimodal histogram for a well-defined threshold selection [16, 35, 36]. This method was used on the complete data set and the cropped data set and not on single slices, as described by Vaitiekunas et al. [15].

This study's main goal was to evaluate the influence of teeth and dental restorations on the gray value distribution, represented in selected threshold levels for bone segmentation, not the comparison of surface models generated by the described threshold selection procedures. Nevertheless, the comparison of both segmentation methods showed that automated and manual threshold selection differed, which can influence the surgical outcome by an over or underestimation of bone quantity and the misinterpretation of bone quality.

The mean gray values in the presented study were lower than the gray values for CBCT in a previous study. However, they ranged among the stated Hounsfield Units for CT [5, 32]. Grey values are not calibrated among CBCT systems, the field of view, voltage, voxel size, and bit value. Therefore, they may not be directly transferred or defined as Hounsfield units [10, 12, 37]. For standardization within the entire cohort, all CBCT data sets were acquired using the exact specifications and the same CBCT device, as recommended for minimizing the influences of the specific CBCT device and acquisition parameters [8, 9].

In all three threshold selection procedures, higher values were found in partially edentulous patients than fully edentulous patients, except in the maxilla in manual segmentation. This influence seems higher in automated threshold selection methods than in manual threshold selection. Gray value differences ranging from 24 to 110 between fully and partially edentulous patients are assumed to influence surface models significantly [34]. Deviations of up to 0.5 mm between bone surfaces were previously reported for gray value adaption of 60 [34]. Although Hounsfield units cannot be directly assigned to CBCT, especially for density measurements, it is widely used in implant planning software for preoperative bone density evaluation [11]. Especially in situations with remaining teeth, this evaluation may be impaired by the correlation of higher gray values and remaining teeth.

To interpret the results, one should be aware of the limitations of this study. No sample size calculation was performed due to the characteristics of a pilot study. This might result in an underpowered analysis. Using this data, sample size calculations can be performed in further studies to provide adequate power. All investigations were performed on a small cohort without calibrating for bone quality and quantity. The study cohort includes patients of each age and potential secondary diagnoses affecting bone density. Nevertheless, these patients represent patients in daily practice for whom the described techniques are applied for computer-aided design and manufacturing. The size of dental restorations was not considered, which is a downside of this study and should be addressed in further investigations.

## Conclusions

Standard gray values do not apply for bone segmentation in CBCT as teeth influence the gray values and surface models resulting from segmentation. The clinical consequence are ill-fitting surgical guides produced on CBCT data and misinterpretation of bone density, both needed for fully guided implant surgery and determination of implant loading protocols. Automated bone segmentation may not be recommended for clinical routine, especially in partially edentulous patients. Every clinician should be aware of the potential over- and underestimation of bone and misinterpretation of bone quality in CBCT imaging in relation to the known artifacts and existing teeth. Automated segmentation methods, considering anatomical and acquisition specifications as shown in this study, could improve surface model generation using CBCT data.

### Supplementary Information

**Additional file 1: Table S1.** Gray values for each segmentation procedure in the maxilla. **Table S2.** Gray values for each segmentation procedure in the mandible.

## Data Availability

The data sets used and/or analyzed during the current study are available from the corresponding author on reasonable request.

## Abbreviations

CBCT: Cone-beam computed tomography
CAD/CAM: Computer-aided design/computer-aided manufacturing
HU: Hounsfield Units
FOV: Field of view
DICOM: Digital Imaging and Communications in Medicine
