# Supplementary material for: The impact of teeth and dental restorations on gray value distribution in cone-beam computer tomography: a pilot study
Source: Int J Implant Dent. 2023 Sep 7;9:27. doi: 10.1186/s40729-023-00493-z (PMC10484826; doi:10.1186/s40729-023-00493-z)
Supplement: Supplementary file 1 — Additional file 1: Table S1. Gray values for each segmentation procedure in the maxilla. Table S2. Gray values for each segmentation procedure in the mandible. [file 40729_2023_493_MOESM1_ESM.docx]

Additional Tables

Additional file 1: Table S1. Gray values for each segmentation procedure in the maxilla.

| Patient | analyzed jaw | Manual threshold selection ROI | Automated threshold complete CBCT | Automated threshold ROI |
| --- | --- | --- | --- | --- |
| 1 | edentulous | 181 | 222 | 72 |
| 2 | edentulous | 458 | 377 | 328 |
| 3 | edentulous | 337 | 323 | 249 |
| 4 | edentulous | 297 | 367 | 287 |
| 5 | edentulous | 233 | 275 | 228 |
| 6 | edentulous | 174 | 251 | 177 |
| 7 | edentulous | 142 | 171 | -18 |
| 8 | edentulous | 157 | 292 | 166 |
| 9 | edentulous | 173 | 257 | 89 |
| 10 | edentulous | 267 | 326 | 238 |
| 11 | partial | 302 | 348 | 279 |
| 12 | partial | 334 | 343 | 292 |
| 13 | partial | 281 | 375 | 369 |
| 14 | partial | 215 | 326 | 258 |
| 15 | partial | 198 | 275 | 288 |
| 16 | partial | 236 | 306 | 311 |
| 17 | partial | 116 | 255 | 174 |
| 18 | partial | 146 | 198 | 94 |
| 19 | partial | 164 | 297 | 294 |
| 20 | Partial | 119 | 254 | 209 |

Additional file 1: Table S2. Gray values for each segmentation procedure in the mandible.

| Patient | analyzed jaw | Manual threshold selection | Auto threshold complete CBCT | Auto threshold ROI |
| --- | --- | --- | --- | --- |
| 21 | edentulous | 338 | 257 | 331 |
| 22 | edentulous | 523 | 367 | 402 |
| 23 | edentulous | 386 | 368 | 375 |
| 24 | edentulous | 163 | 241 | 274 |
| 25 | edentulous | 466 | 292 | 405 |
| 26 | edentulous | 396 | 171 | 163 |
| 27 | edentulous | 267 | 206 | 295 |
| 28 | edentulous | 295 | 255 | 337 |
| 29 | edentulous | 268 | 252 | 322 |
| 30 | edentulous | 353 | 74 | 324 |
| 31 | partial | 293 | 361 | 411 |
| 32 | partial | 559 | 326 | 397 |
| 33 | partial | 382 | 198 | 273 |
| 34 | partial | 531 | 323 | 414 |
| 35 | partial | 462 | 275 | 371 |
| 36 | partial | 589 | 343 | 390 |
| 37 | partial | 503 | 348 | 279 |
| 38 | partial | 500 | 326 | 386 |
| 39 | partial | 552 | 306 | 337 |
| 40 | partial | 392 | 254 | 334 |
